# Nondestructive Evaluation for Hydration and Setting Time of Gypsum Modified Calcium Sulfoaluminate Cement Paste

**DOI:** 10.3390/ma16030920

**Published:** 2023-01-18

**Authors:** Yubin Jun, Yu-Rhee Ahn, Dongho Jeon, Hong Jae Yim

**Affiliations:** 1 Department of Civil and Environmental Engineering, Korea Advanced Institute of Science and Technology, Daejeon 34141, Republic of Korea; 2 Department of Civil Engineering, Pusan National University, Busan 46241, Republic of Korea; 3 Department of Civil and Environmental Engineering, Seoul National University, Seoul 08826, Republic of Korea

**Keywords:** ultrasonic pulse velocity, electrical resistivity, CSA, gypsum, ye’elimite

## Abstract

Calcium sulfoaluminate (CSA) cement is a promising solution for reducing CO_2_ emissions. While previous studies have attempted to investigate the usefulness of CSA cement via various approaches, early age nondestructive evaluations for the setting and hydration of CSA cement mixtures have not been reported. In this study, we measured the ultrasonic pulse velocity and electrical resistivity of early age CSA cement paste. Six types of samples were prepared according to different water-to-solid ratios and different amounts of gypsum. In addition, various microstructural analyses were performed to understand CSA cement hydration with the obtained nondestructive parameters. Consequently, the effect of added gypsum in CSA cement paste was discussed in terms of ye’elimite dissolution and the precipitation of ettringite, and different pore distributions produced by added gypsum were discussed in terms of compressive strength. The 5% addition of gypsum in CSA cement paste enhanced the hydration evolution, such as ettringite, and it can induce the faster setting time up to 6 h and strength development during 24 h.

## 1. Introduction

Ordinary Portland cement has long been used as one of the main construction materials in the construction industry. However, the emission of a large amount of CO_2_ from the calcination of carbonaceous materials and the burning of fuels during the cement manufacturing process increases air pollution, causing environmental problems such as global warming and climate change. The cement industry accounts for approximately 8% of global CO_2_ emissions [1]. With the interest in global environmental issues, recycled concrete, such as mortars with recycled manufactured sand [2], was considered, and there is an increasing need for an alternative to cement to reduce the increasing use of cement. Calcium sulfoaluminate (CSA) cement is a promising solution to reduce CO_2_ emissions [3]. CSA cement is produced by calcination at approximately 200 °C lower than that of Portland cement, which contributes to energy savings and CO_2_ emission reduction during production [4,5,6].

The main mineral phase of CSA cement is ye’elimite ($C_{4}A_{3}\bar{S}$) [7]. Ye’elimite hydrates into monosulfate ($C_{4}{A\bar{S}H}_{12}$) via Reaction (1) in the presence of water only. In the presence of calcium sulfate, the hydration of ye’elimite gives rise to ettringite ($C_{6}A{\bar{S}}_{3}H_{32}$) via Reaction (2).

(1) $$C_{4}A_{3}\bar{S}+18H\to {\;C}_{4}{A\bar{S}H}_{12}+2{\mathrm{AH}}_{3}$$

(2) $$C_{4}A_{3}\bar{S}+2C\bar{S}+38H\to {\;C}_{6}{A\bar{S}}_{3}H_{32}+2{\mathrm{AH}}_{3}$$

Monosulfate ($C_{4}{A\bar{S}H}_{12}$), ettringite ($C_{6}{A\bar{S}}_{3}H_{32}$), and aluminum hydroxide (AH_3_) were precipitated by the dissolution of ye’elimite at early ages (Reactions (1) and (2) [8,9]; cement chemist shorthand notation: C = CaO, A = Al_2_O_3_, $\bar{S}$ = SO_3_, and H = H_2_O). The hydration product of ye’elimite varies depending on the presence or absence of calcium sulfate. This can affect the setting time and mechanical strength [10].

The setting of cement-based materials is a critical point for indicating the phase change from a concentrated suspension to a solid material with mechanical strength. Penetration resistance measurement methods, such as the Vicat method, have been widely used to evaluate the setting time of fresh cement-based materials, such as cement paste, mortar, and concrete [11,12]; however, they hardly reflect the microstructural development in porous materials and have limitations for in situ evaluation. To monitor the degree of cement hydration and microstructural changes, several test methods, such as chemical analysis and nondestructive approaches, have been suggested and reported with regard to different hydration processes owing to various materials and curing conditions [13,14,15].

Among them, nondestructive evaluation methods using ultrasonic wave propagation characteristics (e.g., ultrasonic wave velocity) [16,17] and electrical properties (e.g., electrical resistivity and impedance) [15,18,19,20] have been proposed for the phase change monitoring of cement-based materials, including solid percolation and water depercolation [21]. Previous studies investigated the effect of various materials composed of different mix proportions and admixtures; however, a nondestructive approach for setting and hardening evaluation in fresh-state CSA cement mixtures and the investigation of their physicochemical changes have not been reported. Because CSA cement mixture follows different hydration process than the use of ordinary cement, it requires a more applicable technique to reflect microstructural changes directly. This study attempted to measure the ultrasonic pulse velocity (UPV) and electrical resistivity to monitor the microstructural evolution and evaluate the setting time in CSA cement paste in the presence and absence of different amounts of gypsum. In addition, the hydration products of CSA cement pastes were explored using X-ray diffraction (XRD) and thermogravimetry (TG). Compressive strength tests, mercury intrusion porosimetry (MIP), and scanning electron microscopy (SEM) were conducted to compare the mechanical properties and microstructures of hardened CSA cement pastes.

## 2. Experimental Program

### 2.1. Materials

**Figure 1:**
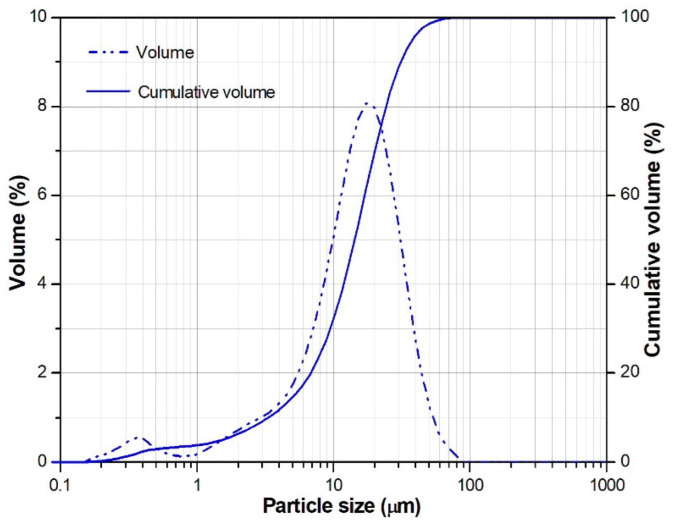
Particle size distribution of CSA cement.

**Figure 2:**
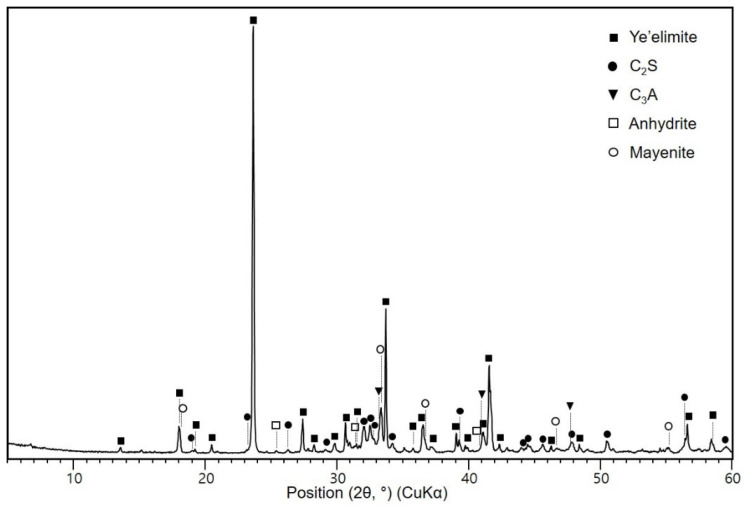
X-ray diffraction pattern of CSA cement. Database code: Ye’elimite (ICDD 33-0256); C_2_S (ICSD 79551); C_3_A (ICSD 151369); Anhydrite (ICSD 068592); Mayenite (ICDD 09-0413).

**Figure 3:**
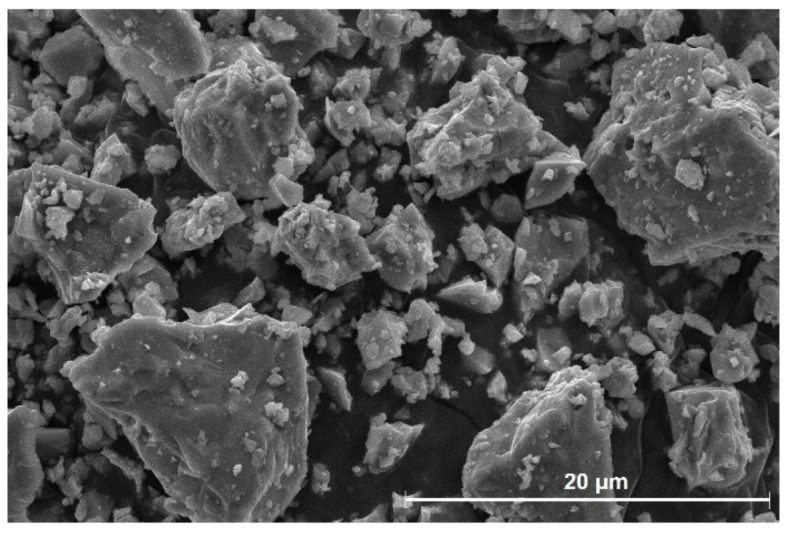
SEM micrograph of CSA cement.

Commercially available CSA cement was used in this study. Its density and Blaine fineness (expressed as the specific surface area of fines) were 2.84 g/cm^3^ and 446 m^2^/kg, respectively. The chemical composition was analyzed using an X-ray fluorescence spectrometer (S8 Tiger, Bruker, Karlsruhe, Germany) and is presented in Table 1. The particle size distribution was measured using a laser scattering particle size distribution analyzer (LA-950, Horiba Ltd., Kyoto, Japan), as illustrated in Figure 1. The CSA cement exhibited a particle size range of ~0.17–78 μm, with a median particle size of 14.3 μm. The physical properties of CSA cement are tabulated in Table 2. The crystalline phases of the CSA cement, identified by XRD analysis, included ye’elimite, C_2_S, C_3_A, anhydrite, and mayenite (Figure 2). The contents of the phases were calculated by Rietveld method using the XRD data of the CSA cement and are presented in Table 3. Figure 3 shows the SEM micrograph of CSA cement particles at ×3000 magnification, which are irregularly shaped with low angularity. Reagent-grade gypsum (CaSO_4_·2H_2_O, calcium sulfate dihydrate) was used as an additive.

### 2.2. Sample Preparation

The CSA cement was replaced with 0, 3, and 5% gypsum by weight. Samples with gypsum contents of 0, 3, and 5 wt. % were labeled as G0, G3, and G5, respectively. The weight ratios of water to solid (w/s) were 0.5 and 0.55, denoted as A and B, respectively. Based on preliminary experiments, the addition amount of gypsum and the w/s were determined, considering workability and rheological properties. The mixture proportions of the samples are listed in Table 4.

All mixtures were mixed for 2 min using a planetary mixer (HJ-1150, Seoul, Republic of Korea), here the capacity of used mixer is 4.7 L with 140 rpm, and cast in molds for the ultrasonic pulse velocity and electrical resistivity measurements. The fresh pastes were cast into 40 mm × 40 mm × 40 mm molds for compressive strength tests and 25 mm × 25 mm × 25 mm molds for XRD, TG, and MIP measurements. Temperature and relative humidity and curing conditions for testing were maintained at 25 °C and 50%, respectively.

### 2.3. Test Method

#### 2.3.1. UPV

**Figure 4:**
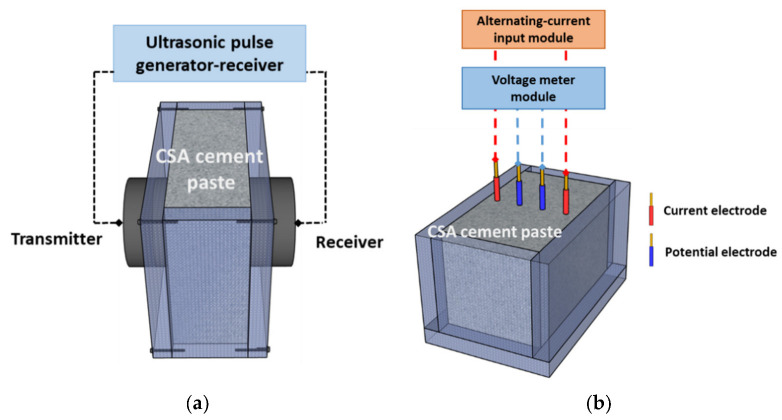
Experimental setup for nondestructive evaluation method 24 h after mixing of CSA pastes: (**a**) ultrasonic velocity measurement system, and (**b**) electrical resistivity measurement system.

Figure 4a shows the experimental setup of the through-transmission method for UPV measurements in the CSA cement pastes. The UPV was primarily determined by the propagation time through the shortest path of the solid network in the paste mixture, and the propagating velocity might be enhanced with increased percolation paths owing to coagulation and aggregation of solid particles. This implies that UPV monitoring can represent the hardening and evolution of the elastic modulus of cementitious mixtures. This study monitored the early age CSA cement paste for 24 h at a time interval of 10 min. For UPV measuring equipment, the ultrasound device Pundit Lab+ (Proceq, Schwerzenbach, Switzerland) was used. This portable device was used for a pulse input of 10 V, and two transducers for generating and receiving were attached to the sample surface and fixed using a fabricated mold, where the center frequency of the transducers was 54 kHz. A cuboid-shaped mold with dimensions of 40 mm × 40 mm × 160 mm was fabricated using non-conducting material (polyethylene) for mixture casting and testing. Each measurement was an averaged result of ten acquired signals to decrease the noise effect. Accordingly, the UPV was calculated based on the propagation path of 40 mm and the obtained time of flight of ultrasound by the time-domain difference of both the input and output signals. The monitoring results were averaged for three duplicate samples under the same curing conditions.

#### 2.3.2. Electrical Resistivity

Figure 4b shows the measurement setup for the electrical resistivity of CSA cement paste samples. Wenner’s four-electrode method was used to monitor the electrical resistivity over 24 h. This method has been adopted to measure the early age state in cementitious materials; here, the changed electrical conductivity is determined by ion transport through the water-filled porosity in the fresh state CSA cement paste. This setup was set to four electrodes with a spacing of 20 mm, and two outside electrodes and two inside electrodes were used for the current and potential measurements, respectively. Copper electrodes with a diameter of 1.78 mm were used and inserted in the center of the sample. A waveform generator (National Instrument 9263, Austin, TX, USA) was used to generate a sinusoidal potential at the outer current electrodes with a voltage amplitude of 10 V at a frequency of 500 Hz. An electric field was created inside the samples from these two electrodes, and an alternating-current input module (National Instrument 9227, Austin, TX, USA) at the outer set of electrodes measured the resultant current. In addition, a voltmeter (National Instrument 9222, Austin, TX, USA) at the inner set of electrodes simultaneously measured the resultant potential difference. From these measurement results, the electrical resistance was obtained using Ohm’s law (R=V/I*)* and the electrical resistivity was calculated based on the geometric relation ρ=2πaR (a: electrode spacing). Monitored electrical resistivity for 24 h with intervals of 10 min were averaged over three duplicate samples; the tests were performed in a cuboid-shaped mold with dimensions of 40 mm × 60 mm × 90 mm that was also fabricated using a non-conducting material (polyethylene).

#### 2.3.3. Pretreatment for XRD, TG, MIP, and SEM

**Figure 5:**
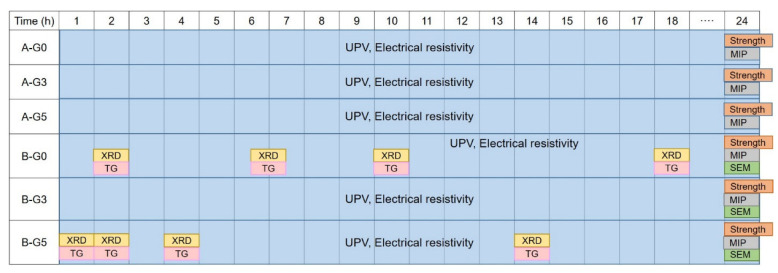
Selection and description of test cases according to a test purpose.

For the XRD and TG experiments, 25 mm cubic paste samples were crushed and ground to a particle size of <75 μm. For the MIP and SEM tests, the cores of the samples were cut into 5 mm × 5 mm × 5 mm blocks using a low-speed diamond saw. All prepared specimens were immersed in an isopropanol solution to stop hydration and dried prior to use. The characteristics of the hydration products at a specific hydration time of a representative sample, reflected by the UPV and electrical resistivity tests, were evaluated in this study (Figure 5).

#### 2.3.4. XRD

XRD was conducted using a high-resolution powder diffractometer (SmartLab, Rigaku, Tokyo, Japan) with Cu Kα radiation (λ = 1.5406 Å). The scanning rate was 2° per minute, ranging from 5 to 60° (2θ). The XRD patterns were analyzed using the Inorganic Crystal Structure Database (ICSD) and International Center for Diffraction Data (ICDD).

#### 2.3.5. TG

TG measurements were performed using a TG 209 F1 Libra thermogravimetric analyzer (Netzsch, Selb, Germany). The heating rate was 10 °C/min, and the temperature was increased to 1000 °C in a nitrogen environment. TG and differential TG (DTG) curves are presented.

#### 2.3.6. MIP

MIP was performed using an AutoPore IV 9500 (Version 2.01) device (Micromeritics, Norcross, GA, USA). The maximum mercury intrusion pressure is 60,000 psi (414 MPa).

#### 2.3.7. Compressive Strength

For each mix proportion, the 24 h compressive strength of the 40-mm cube specimens was determined. The tests were performed at a displacement rate of 1 mm/min. The average of the three values was recorded as the compressive strength result for each test.

#### 2.3.8. SEM

Microstructural characterization of the hardened sample was conducted using a field-emission scanning electron microscope (Hitachi SU-70, Tokyo, Japan). Secondary electron (SE) images of the saw-cut surfaces of the samples were obtained. The sections were coated with platinum (Pt) prior to SEM observation.

## 3. Results and Discussion

### 3.1. UPV

**Figure 6:**
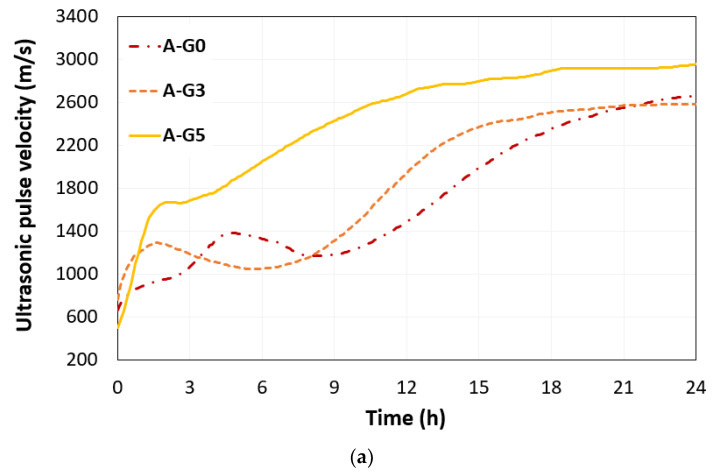

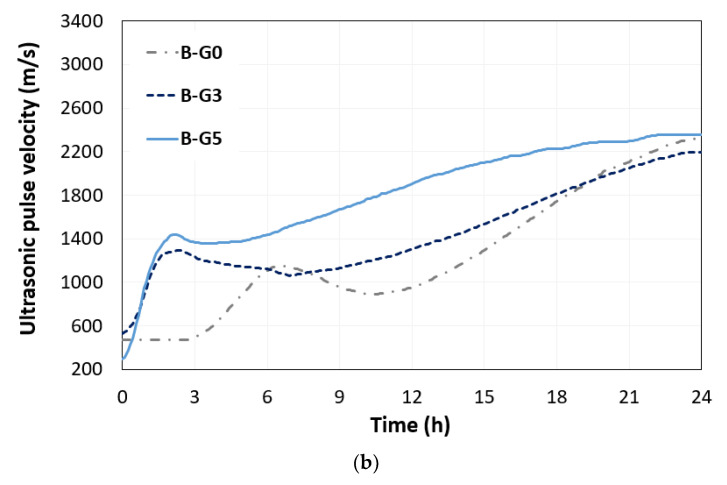
UPV measurements of early-age CSA cement paste with the different gypsum contents of 0% (G0), 3% (G3), and 5% (G5) under different water-to-binder ratios (**a**) 0.5 and (**b**) 0.55.

The UPVs monitored for 24 h in all CSA cement pastes were compared with different gypsum amounts, as shown in Figure 6a,b for w/s of 0.5 and 0.55, respectively. The increasing trend of UPV reflects the microstructural evolutions in early age CSA cement paste caused by the hydration process, and the decreased UPV can be caused by the microstructural changes such as increased porosity or hydration transformation. In Figure 6b, the B-G0 sample shows that agglomerated CSA cement particles led to the development of a solid network and an increase in UPV. However, from 6.5 h, the UPV began to decrease until 10 h. This result indicates that the dissolution rate of the CSA cement particles was faster than that of hydrate generation, and the solid network thickness decreased. In samples B-G3 and B-G5, the added gypsum crystal accelerated the solid network and induced faster setting and hardening. This phenomenon resulted in a rapid increase in the UPV, which reached approximately 1400 m/s before 3 h after mixing. The added gypsum also decelerated the dissolution rate of CSA cement particles. This trend was also observed in group A. In sample group A, the increasing rate of UPV was reduced and converged at approximately 18 h in A-G3 and A-G5, and sample group B with higher water content also showed that the UPV converged at approximately 24 h in the gypsum-added mixtures (B-G3 and B-G5). The convergence time of the UPV denotes the fully connected solid-phase network in the mixture. The added gypsum led to the early hydration and early set of solid-phase networks in the short path for the propagation of ultrasonic waves, and this trend was enhanced in mixtures with lower w/s.

### 3.2. Electrical Resistivity

**Figure 7:**
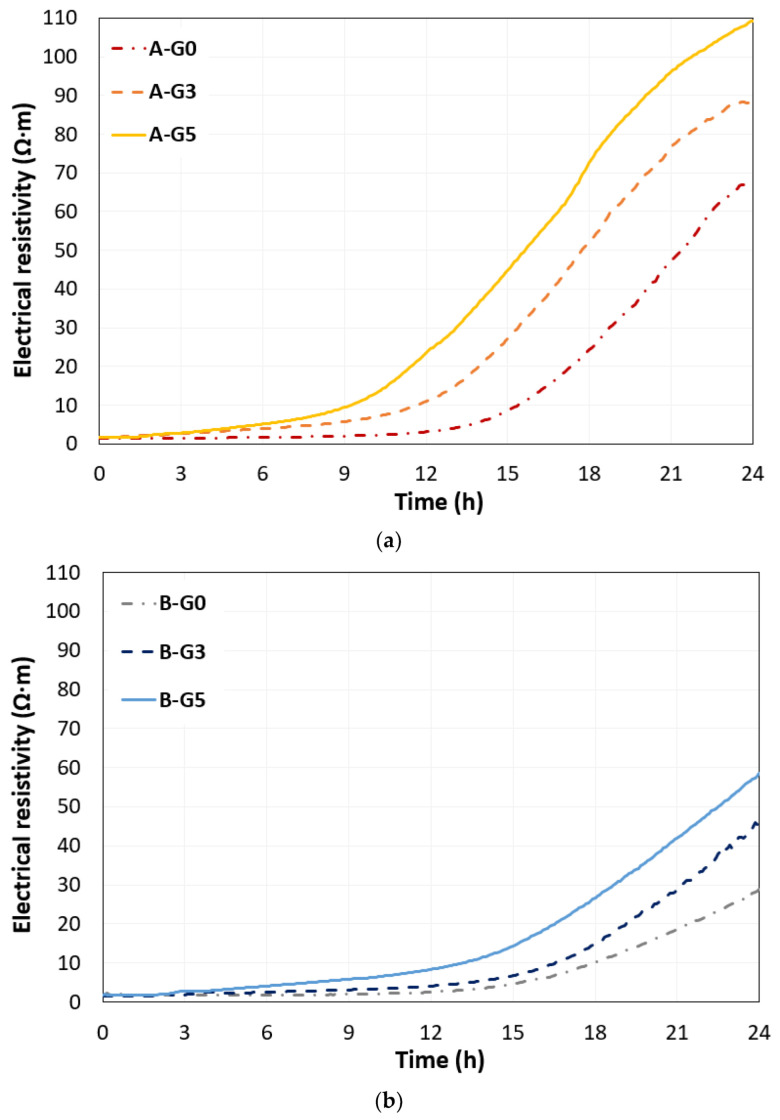
Electrical resistivity measurements of early-age CSA cement paste with the different gypsum contents of 0% (G0), 3% (G3), and 5% (G5) under different water-to-binder ratios (**a**) 0.5 and (**b**) 0.55.

Figure 7a,b show the electrical resistivities of both the sample groups. In all mixtures, the initial value of the electrical resistivity was approximately 2 Ω·m, and a few hours later, the electrical resistivity remarkability increased with the microstructural evolution. The increased point can be defined as the rising time of electrical resistivity. A previous study identified that the initial value of electrical resistivity is determined by the initial water content and its dispersed solid particles in a fresh cementitious mixture, and the rising time of electrical resistivity as a non-destructive parameter reflects the time of the depercolated water-filled pore network [21]. This nondestructive parameter can be used as a setting time indicator based on microstructural changes owing to the generation of specific hydrates. Accordingly, in this study, the rising time was determined to be four times that of the initial electrical resistivity. The obtained rising time for samples A-G0, A-G3, and A-G5 were 14.8 h, 10.5 h, and 8.5 h, respectively, and for samples B-G0, B-G3, and B-G5 were 17 h, 15.7 h, and 11.7 h, respectively. The rising time or change in electrical resistivity can reflect the change in water-filled microstructures, and this change primarily occurred after the UPV was changed. The electrical resistivity rising time decreased as the water content decreased and gypsum amount increased. After the rising time, the electrical resistivity and its increased rate (Ω·m/h) represent the reduced water-filled porosity in the CSA cement paste. At 24 h after mixing, the obtained electrical resistivities were 67, 88, and 109 Ω·m for samples A-G0, A-G3, and A-G5, respectively, and 28, 45, and 57 Ω·m for samples B-G0, B-G3, and B-G5, respectively. These results of electrical resistivity were difficult to directly correlate with the compressive strength at 24 h; however, it can be concluded that added gypsum enhances the number of needle-type hydrate generations, such as ettringite, and it led to a higher electrical resistivity but hardly influenced the mechanical strength of the materials.

### 3.3. XRD and TG Analysis

**Figure 8:**
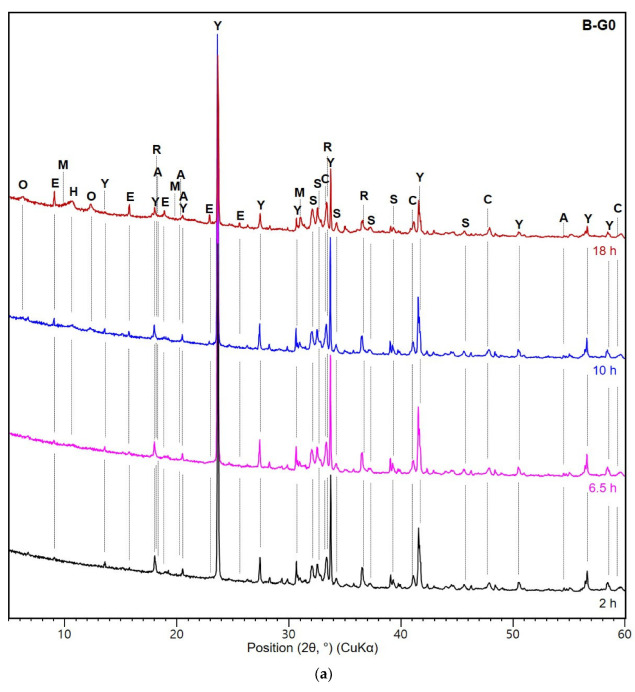

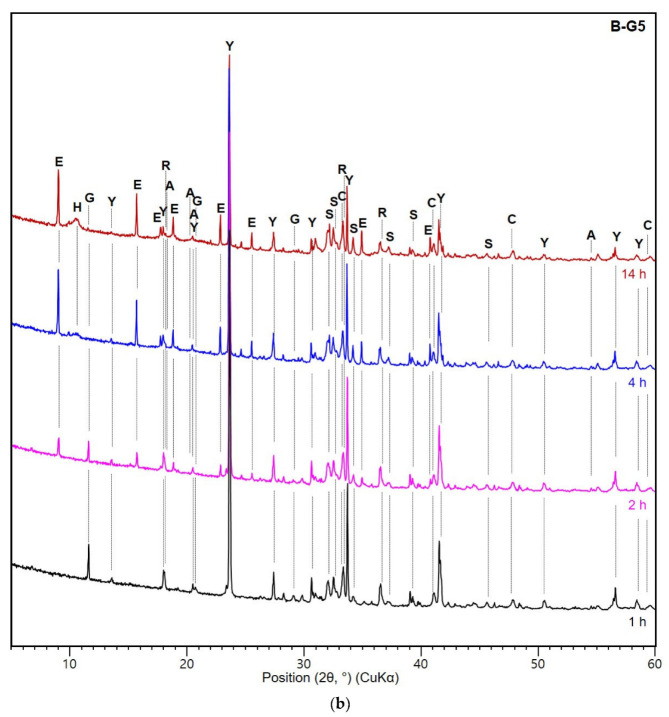
XRD patterns of (**a**) B-G0 and (**b**) B-G5 at each curing time. O: CAH_10_; E: ettringite; M: monosulfate; H: hydroxy-AFm; Y: ye’elimite; R: mayenite; A: AH_3_; S: C_2_S; C: C_3_A; G: gypsum.

The XRD patterns for the 2, 6.5, 10, and 18 h hydrated B-G0 pastes and the 1, 2, 4, and 14 h hydrated B-G5 pastes are shown in Figure 8. In B-G0 (Figure 8a), ettringite and AH_3_ were identified at 2 h, and their peak intensities increased with time. They were formed by the hydration of ye’elimite with anhydrite (originally present in raw CSA). With increasing hydration time (<18 h), anhydrite was almost completely consumed and ye’elimite remained unhydrated. At 6.5 h, hydroxy-AFm was observed. A peak appeared at approximately 12.3° 2θ between 10 and 18 h. This is attributed to the presence of CAH_10_. CAH_10_ reportedly occurs when the anhydrite to ye’elimite ratio is low [22]. At 18 h, monosulfate was observed, which was attributed to the reaction between ye’elimite and water owing to the exhaustion of anhydrite [23].

When gypsum was added to the CSA cement (Figure 8b), ettringite was observed after 2 h, as in the B-G0 sample. The ettringite peaks appeared simultaneously with or without the addition of gypsum. However, B-G5 produced strong ettringite peaks owing to the supply of sulfate. As the hydration of the CSA cement proceeded, ettringite was formed from the consumption of ye’elimite and gypsum. Gypsum was rapidly consumed between 2 and 4 h. After 4 h, a trace amount of gypsum was still present, indicating the absence of monosulfate formation. After 4 h, hydroxy-AFm was identified, which increased with time. In B-G0 and B-G5, a little change was observed in the peak intensities of the other components of CSA cement (i.e., mayenite, C_3_A, and C_2_S) with hydration time.

**Figure 9:**
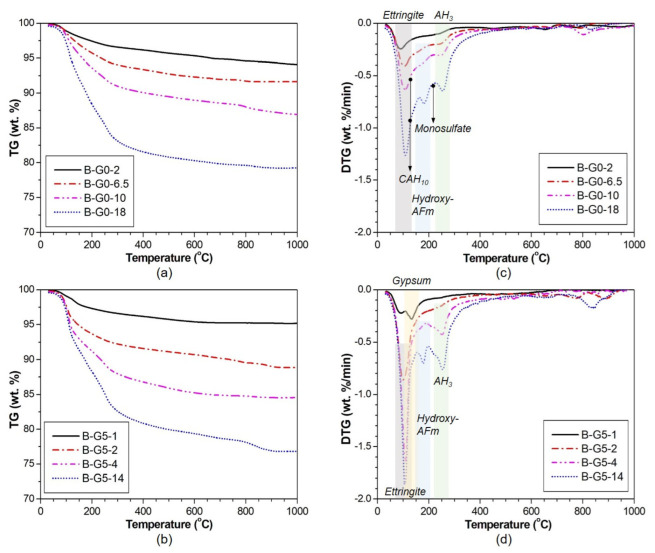
TG and DTG curves for B-G0 and B-G5 at each curing time. (**a**) TG curve of B-G0; (**b**) TG curve of B-G5; (**c**) DTG curve of B-G0; (**d**) DTG curve of B-G5.

Figure 9 shows TG and DTG curves of B-G0 and B-G5 samples with curing time. The weight losses of hydration products primarily occurred below 300 °C. In both samples, large peaks appeared from 80 to 130 °C [24] in the DTG curves owing to the decomposition of ettringite. The DTG peak size of ettringite was noticeably larger in B-G5 when compared to that of B-G0. This observation is consistent with the XRD result. The weight loss around 250–300 °C [23] showed the decomposition of AH_3_. The weight loss near 195 °C [25] was attributed to the decomposition of hydroxyl-AFm.

In B-G0, the weight losses near 112–130 °C [26] and 210–220 °C [25] are related to the presence of CAH_10_ and monosulfate, respectively. In B-G5, the peaks between 100 and 200 °C [27] indicated the residual presence of gypsum.

### 3.4. Compressive Strength and MIP Results

**Figure 10:**
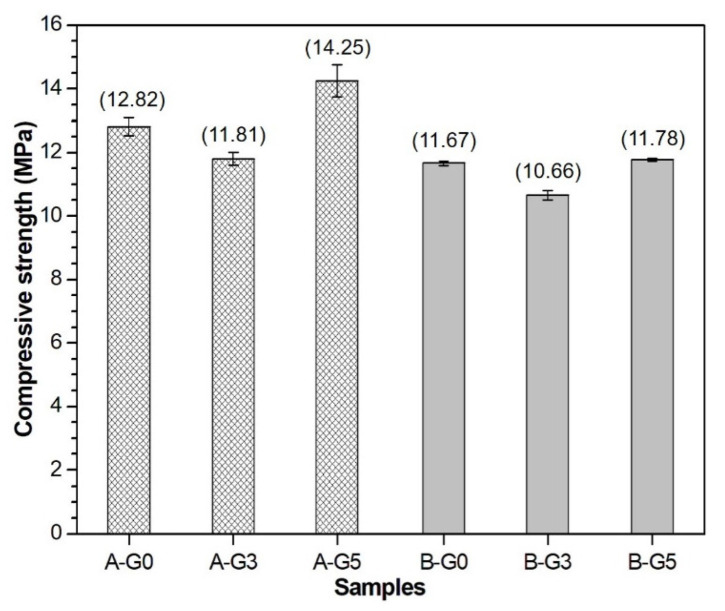
24 h compressive strength of CSA cement paste containing 0% (G0), 3% (G3), and 5% (G5) gypsum. A: w/s ratio of 0.5; B: w/s ratio of 0.55.

All the compressive strength testing results of the CSA cement pastes at 24 h are presented in Figure 10. As the data indicate, a higher w/s ratio of 0.55 resulted in a lower compressive strength for the CSA cement pastes. Regardless of the w/s ratio, the addition of 3% gypsum decreased the strength of CSA cement paste. However, when the gypsum content was increased to 5%, the strength increased to a higher level than that of the sample without gypsum at a w/s ratio of 0.5. According to the w/s ratio and gypsum replacement, the difference in compressive strength also follows the UPV results at 24 h. Adding 3% gypsum reduces the UPV at 24 h compared to the result in the sample without gypsum, but the addition of 5% gypsum enhances the UPV in companies with increased compressive strength.

**Figure 11:**
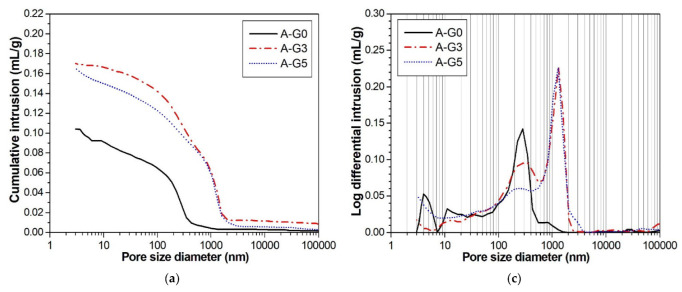

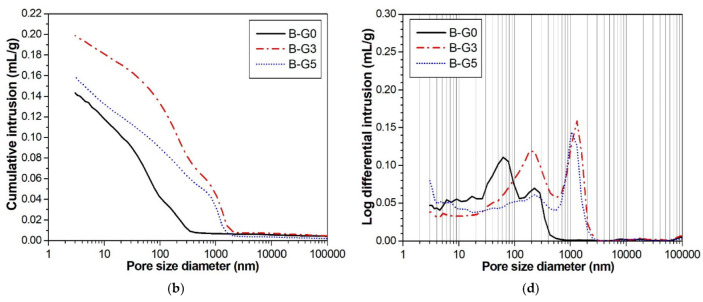
Pore size distribution curves of CSA cement pastes containing 0% (G0), 3% (G3), and 5% (G5) gypsum at 24 h. A: w/s ratio of 0.5; B: w/s ratio of 0.55. (**a**,**b**) for cumulative distribution; (**c**,**d**) for log differential distribution.

MIP testing illustrated how the use of gypsum in the CSA cement affected the pore size distribution, as shown in Figure 11. Regardless of the w/s ratio, the total porosity notably increased when 3% gypsum was added (G3). Compared with the absence of gypsum (G0), the difference in the hydration products was due to the formation of ettringite. This was probably responsible for the higher porosity and lower strength development of G3. It has been reported that ettringite formation can either contribute to an increase in strength owing to pore filling or cause a reduction in strength owing to increased porosity [28]. However, the addition of 5% gypsum (G5) reduces the porosity of G3. As shown in Figure 11c,d, the reduction in porosity is mainly attributed to the decrease in pores with sizes between 30 and 700 nm. It is likely that the density of ettringite in G5 is higher than that in G3. This decrease in porosity resulted in a higher compressive strength in G5.

### 3.5. SEM

**Figure 12:**
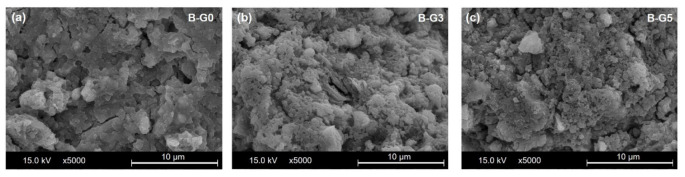
Microstructure of samples measured at 24 h by SEM in SE image mode. (**a**) B-G0 (no gypsum addition); (**b**) B-G3 (addition of 3% gypsum); (**c**) B-G5 (addition of 5% gypsum).

Figure 12 shows SE images of samples B-G0, B-G3 and B-G5 after 24 h of hydration. Compared with B-G0 (no gypsum addition), the villous spherical particles were observed in the B-G3 (addition of 3% gypsum) and B-G5 (addition of 5% gypsum) samples. Needle-like crystals, known as ettringite [29], were not found within prepared cross sections of the test specimens for SEM observation. The microstructure for CSA cement matrix was evidently compacted owing to the addition of gypsum. It can be concluded that the addition of gypsum can affect the compactness of matrix in CSA.

## 4. Conclusions

(1) The amount of added gypsum influenced the hydration and microstructural evolution of the CSA cement paste. The addition of 3% and 5% gypsum to the CSA cement led to an early set of solid-phase networks in the short path for the propagation of ultrasonic waves.
(2) The addition of 5% gypsum in CSA cement paste with w/s of 0.5 promoted the growth of ettringite crystals, up to 6 h. This phenomenon was identified by the changed UPV including converged time and the obtained rising time by monitored electrical resistivity during 24 h.
(3) The 5% addition of gypsum induces a beneficial effect for strength development. A relatively dense structure is formed in the hydration products.
(4) Below a gypsum content of 5% in the CSA cement paste, the total porosity increased, and the compressive strength decreased.
(5) A lower water-to-solid ratio resulted in a higher compressive strength for the CSA cement pastes regardless of the addition of gypsum. In addition, the addition of a certain amount of gypsum to the CSA cement can lead to a high production of ettringite but hardly influences the early strength of the CSA cement paste.

## Figures and Tables

**Table 1:** Chemical composition (oxides in wt.%) of CSA cement.

CaO	SiO_2_	Al_2_O_3_	MgO	SO_3_	Fe_2_O_3_	K_2_O	Na_2_O	P_2_O_5_	TiO_2_	LOI
44.73	7.58	33.51	1.31	7.76	2.07	0.60	0.33	0.13	1.59	0.55

**Table 2:** Physical properties of CSA cement.

Density (g/cm^3^)	Blaine Fineness (m^2^/kg)	Median Particle Size (μm)
2.84	446	14.3

**Table 3:** Phase composition of CSA cement as determined by Rietveld analysis of XRD data.

Phase	Ye’elimite	C_2_S	C_3_A	Anhydrite	Mayenite
wt.%	63.9	18.8	9.6	0.4	7.3

**Table 4:** Mixture proportions of paste samples.

Sample Label	w/s	CSA (%)	Gypsum (%)
A-G0	0.50	100	0
A-G3		97	3
A-G5		95	5
B-G0	0.55	100	0
B-G3		97	3
B-G5		95	5

## Data Availability

All data relevant to the study are included in the article.
